# Image dataset on the Chinese medicinal blossoms for classification through convolutional neural network

**DOI:** 10.1016/j.dib.2021.107655

**Published:** 2021-12-01

**Authors:** Mei-Ling Huang, Yi-Xuan Xu, Yu-Chieh Liao

**Affiliations:** Department of Industrial Engineering & Management, National Chin-Yi University of Technology, Taichung, Taiwan

**Keywords:** Chinese medicinal blossom, Classification, Data augmentation, Deep learning

## Abstract

Tree blossoms have been widely used on the prevention and treatment of a variety of diseases in traditional Chinese medicine for thousand years [1,2]. The growth of flowers is not only for their ornamental value, but also for nutritional, medicinal, cooking, cosmetic and aromatic properties. They are a rich source of many compounds, which play an important role in various metabolic processes of the human body [3]. Edible flowers can promote the global demand for more attractive and delicious food, and can improve the nutritional content of gourmet food [4]. Flowers are beneficial for anti-anxiety, anti-cancer, anti-inflammatory, antioxidant, diuretic and immune-modulator, etc. It is very important to identify edible flowers correctly, because only a few are edible [5].

The shapes or colors of different flowers may be very similar. Visual evaluation is one of the classification methods, but it is error-prone and time-consuming [6]. Flowers are divided into flowers from herbaceous plants (flower) and flower trees (blossom). Now there is a public herbaceous flower dataset [7], but lack of dataset for Chinese medicinal blossoms. This article presents and establishes the dataset for twelve most commonly and economically valuable blossoms used in traditional Chinese medicine. The dataset provide a collection of blossom images on traditional Chinese herbs help Chinese pharmacist to classify the categories of Chinese herbs. In addition, the dataset can serve as a resource for researchers who use different algorithms of machine learning or deep learning for image segmentation and image classification.

## Specifications Table

**Table:** 

Subject	Agricultural Sciences, Computer Science
Specific subject area	Image processing, Image identification, Image classification, computer vision
Type of data	Images
How data were acquired	Blossom images were captured by Google search.
Data format	Raw digital image (JPG format)
Parameters for data collection	Both close-up photography and telephoto images for each category were collected. Blurred images were deleted.
Description of data collection	Images of Chinese medicinal blossoms were collected and classified into twelve categories.
Data source location	Institution: National Chin-Yi University of Technology City: Taichung Country: Taiwan Latitude 24.1450556 and Longitude 120.73011
Data accessibility	Repository name: Chinese medicinal blossom-dataset [8] Data identification number: 10.17632/r3z6vp396m.1 Mendeley Data, V1, https://doi.org/10.17632/r3z6vp396m.1

## Value of the Data

• The dataset provide a collection of blossom images on traditional Chinese herbs help Chinese pharmacist to classify the categories of Chinese herbs.
• This dataset can be used not only as an atlas of botany, but also as a training material for Chinese medicine courses.
• This dataset contribute the expansion of blossom images on traditional Chinese herbs.
• Blossom image data help researchers to understand the performance of new algorithms for object detection and image segmentation.

## 1 Data Description

The blossom images of traditional Chinese medicinal herbs were captured by Google search. The images were divided into twelve categories: (1) Syringa, (2) Bombax malabarica, (3) Michelia alba, (4) Armeniaca mume, (5) Albizia julibrissin, (6) Pinus massoniana, (7) Eriobotrya japonica, (8) Styphnolobium japonicum, (9) Prunus persica, (10) Firmiana simplex, (11) Ficus religiosa and (12) Areca catechu. The dataset uploaded to Mendeley is arranged in twelve folders named by blossom categories.

**Fig. 1:**
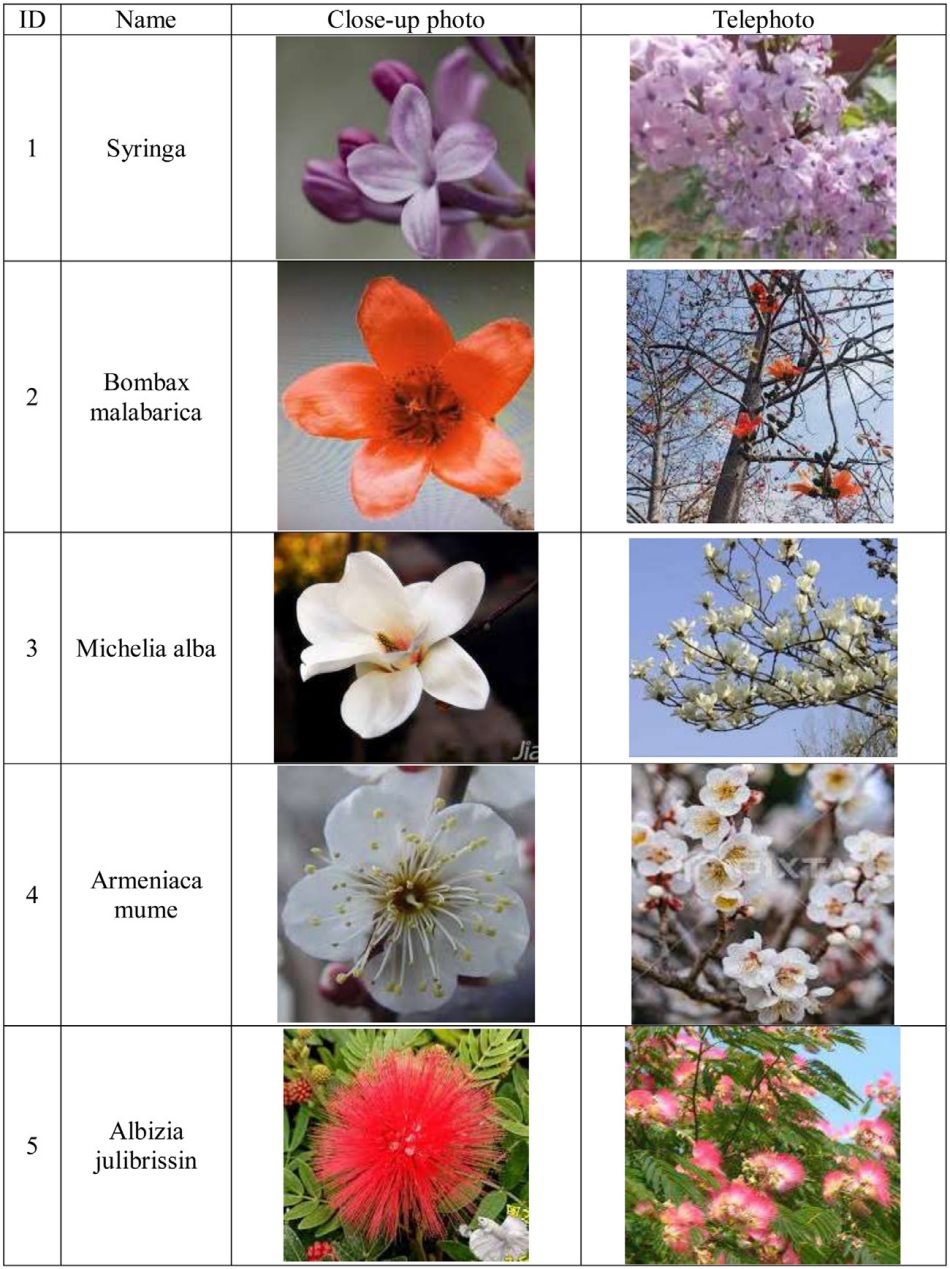

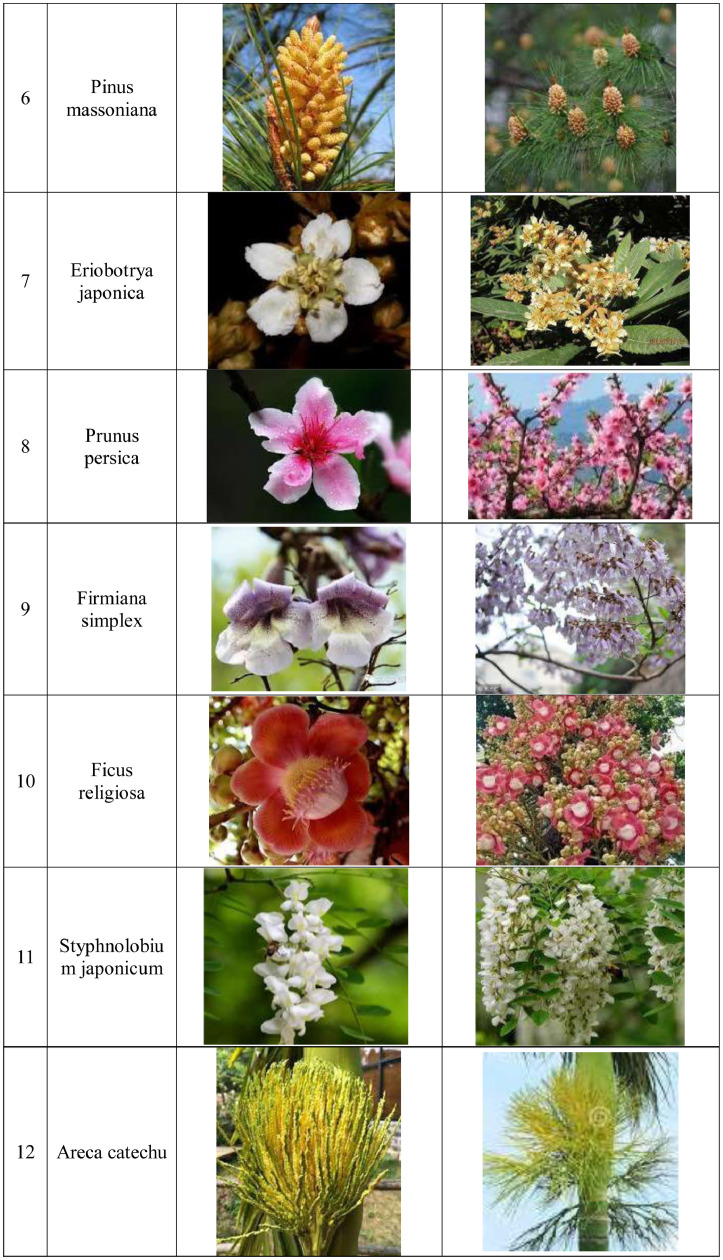
Examples of Chinese medicine blossom categories.
(2) The nomenclature used in the name of the images describes the category, image number in parenthesis, data augmentation method, and image format. For example, the file name “1 (1).JPG” is the first image for the first category “Syringa”; the file name “12 (2).JPG_brighter.jpg” is the second image for the twelfth category “Areca catechu” with augmentation executed by increasing the image brightness.

(1) The number of original images was 1716. Fig. 1 shows examples of the original blossom images for twelve Chinese medicinal herbs. There are both close-up photography and telephoto images for each category.

## 2 Experimental Design, Materials and Methods

**Fig. 2:**
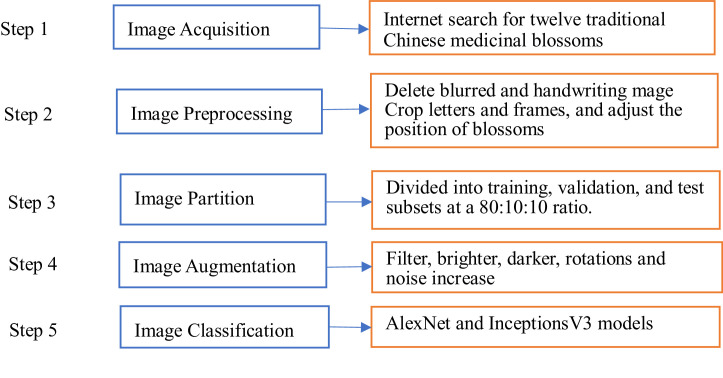
Data processing steps.

Fig. 2 shows data processing steps: image acquisition, image preprocessing, image partition, image augmentation, and image classification as follows.

### 2.1 Image acquisition

Of all the 57 types flower Chinese herbal medicines, there are 12 trees, 9 shrubs, 8 small trees, and 29 herbs. This study selects and establishes the dataset for twelve most commonly and economically valuable tree blossoms used in traditional Chinese medicine. Blossom images were captured through public dataset, personal blog, and government website, etc.

### 2.2 Image preprocessing

We evaluated the blossom images by cropping letters and frames, deleting handwriting and blurred images, centering the blossoms, and adjusting the length and width. The number of images in each category is outlined as follows: (1) Syringa, 191; (2) Bombax malabarica, 172; (3) Michelia alba, 122; (4) Armeniaca mume, 236; (5) Albizia julibrissin, 222; (6) Pinus massoniana, 87; (7) Eriobotrya japonica, 115; (8) Styphnolobium japonicum, 213; (9) Prunus persica, 89; (10) Firmiana simplex,75; (11) Ficus religiosa 126; and (12) Areca catechu, 68. The image file size is not equal, and the image format is in JPG.

### 2.3 Image partition

We amassed a total of 1716 original images in twelve categories. The images were randomly chosen to be divided into training, validation, and test subsets at 80:10:10 ratio for each category. For example, the numbers of training, validation, and test images for Syringa are 153, 19, and 19, respectively. The total number of original images for training, validation, and test subsets were 1376, 170 and 170, respectively.

### 2.4 Image augmentation

**Fig. 3:**
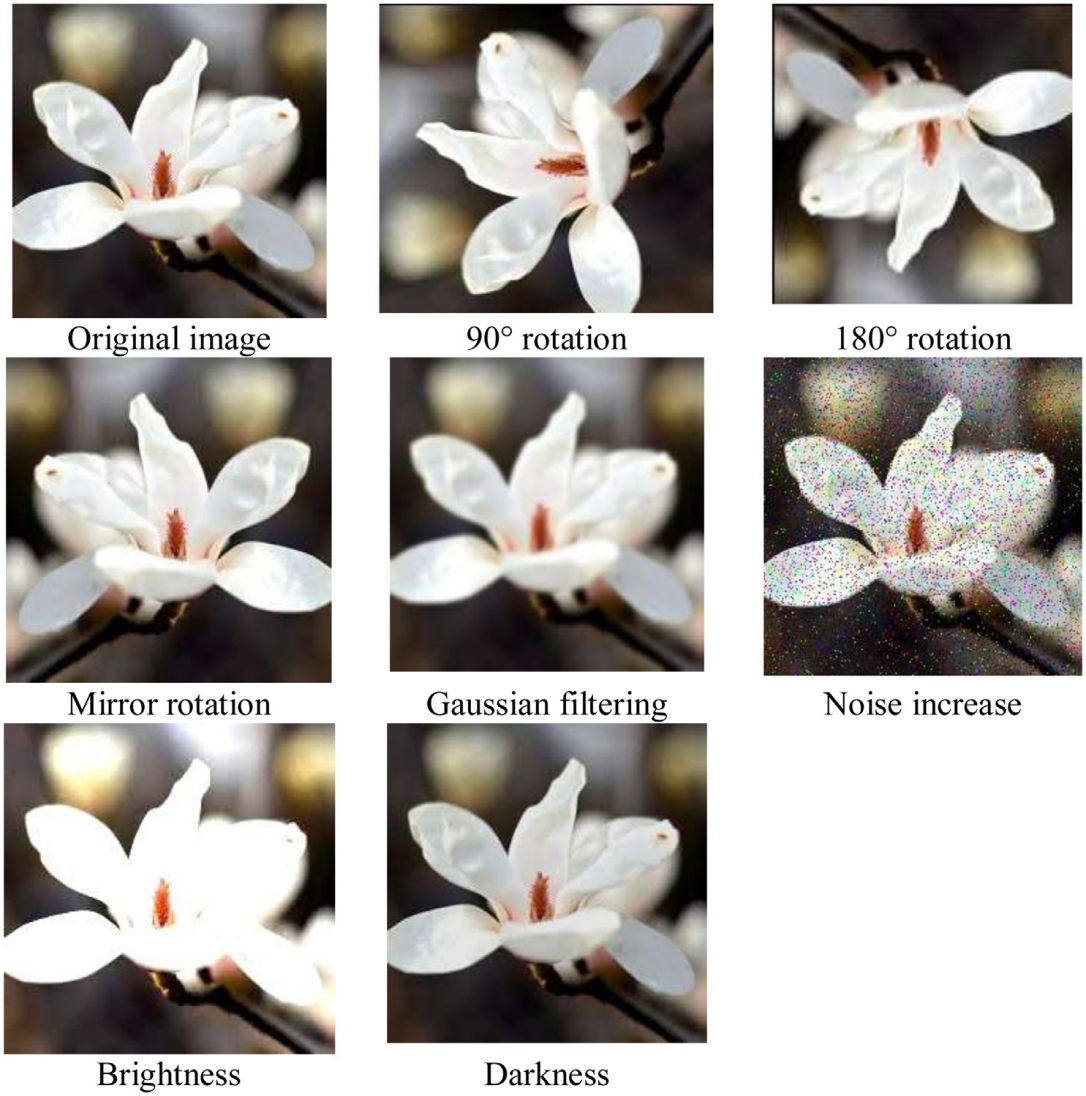
Example of data augmentation.

**Fig. 4:**
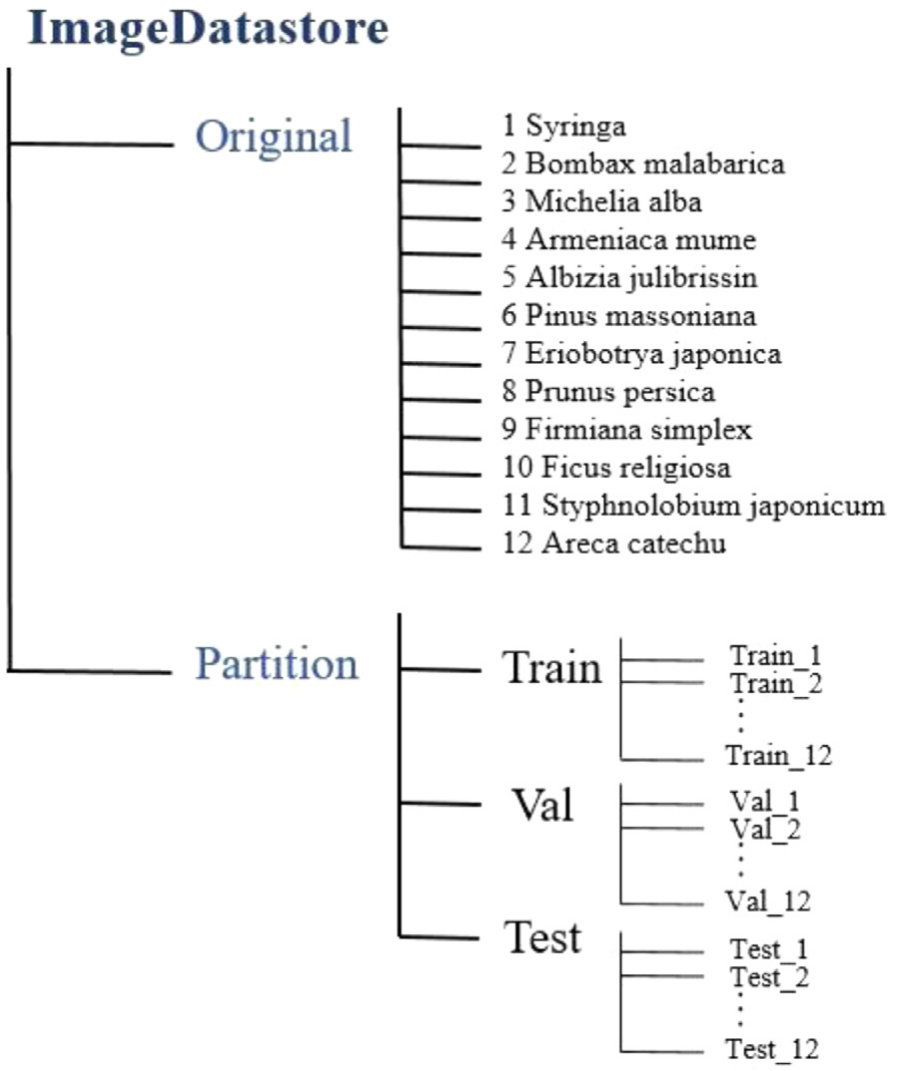
Architecture diagram of dataset.

Data augmentation creates image diversity to enhance performance of classification models. There are many augmentation methods [9], and the benefits may differ from augmentation methods and data characteristics. We select Gaussian filtering, image brightness augmentation, image brightness reduction, mirror rotation, noise increase, 90° rotation, and 180° rotation methods; eight methods in total. Data augmentation was applied in the training and validation datasets. Images were increased to eight times. Fig. 3 shows an example of the original image and the images obtained after data augmentation. Table 1 presents the number of training, validation, and test images before and after data augmentation. Fig. 4 represents the architecture of the dataset.

**Table 1:** Number of images before and after data augmentation.

		Original				After Data Augmentation			
ID	Name	Train	Val	Test	Total	Train	Val	Test	Total
1	Syringa	153	19	19	191	1224	152	19	1395
2	Bombax malabarica	138	17	17	172	1104	136	17	1257
3	Michelia alba	98	12	12	122	784	96	12	892
4	Armeniaca mume	188	24	24	236	1504	192	24	1720
5	Albizia julibrissin	178	22	22	222	1424	176	22	1622
6	Pinus massoniana	70	9	8	87	560	72	8	640
7	Eriobotrya japonica	92	11	12	115	736	88	12	836
8	Prunus persica	171	21	21	213	1368	168	21	1557
9	Firmiana simplex	72	9	8	89	576	72	8	656
10	Ficus religiosa	60	7	8	75	480	56	8	544
11	Styphnolobium japonicum	101	13	12	126	808	104	12	924
12	Areca catechu	55	6	7	68	440	48	7	495

Total		1376	170	170	1716	11008	1360	170	12538

### 2.5 Image classification

**Fig. 5:**
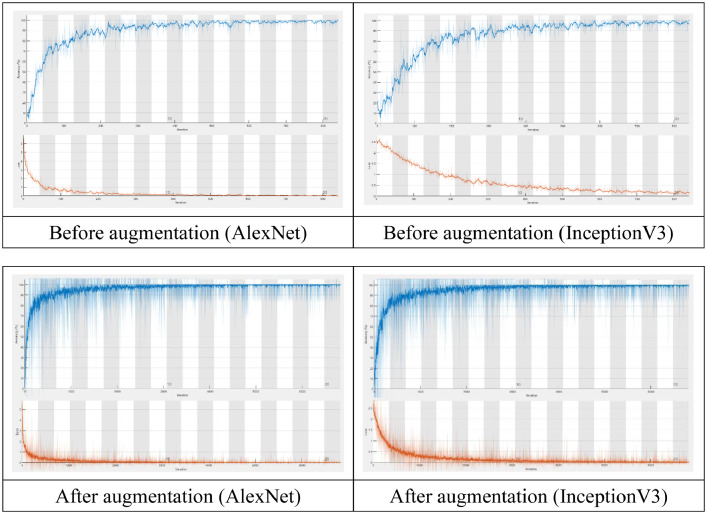
Training curves.

CNN models are the most commonly used for image classification. We selected AlexNet and InceptionV3 models to identify the categories for twelve traditional Chinese medicinal blossoms. Krizhevsky et al. [10] proposed the AlexNet model in 2012. The AlexNet model architecture exhibits eight layers; the first five layers are convolutional layers and the last three layers are fully connected layers. To be more computational efficient, techniques commonly used in InceptionV3 include factorized convolutions, regularization, dimension reduction, and parallelized computations. Tables 2 and 3 showed the results of these two classification models for the datasets before and after data augmentation. Before data augmentation, the accuracy, precision, recall, F1-score, and training time of AlexNet were 93.57%, 92.98%, 94.52%, 93.62%, and 0 h 1 min 17 s, respectively; the accuracy, precision, recall, F1-score, and training time of InceptionV3 were 89.18%, 88.21%, 90.06%, 88.79%, and 0 h 8 min 14 s, respectively. After data augmentation, the accuracy, precision, recall, F1-score, and training time of AlexNet were 98.53%, 98.41%, 98.50%, 98.45%, and 0 h 9 min 26 s, respectively; the accuracy, precision, recall, F1-score, and training time of InceptionV3 were 98.61%, 98.61%, 98.55%, 98.58%, and 1 h 5 min 51 s, respectively. Fig. 5 represents the training curves for the two models for dataset before and after data augmentation.

**Table 2:** Before data augmentation.

	Accuracy	Precision	Recall	F1-score	Time
AlexNet	93.57%	92.98%	94.52%	93.62%	00:01:17
InceptionV3	89.18%	88.21%	90.06%	88.79%	00:08:14

**Table 3:** After data augmentation.

	Accuracy	Precision	Recall	F1-score	Time
AlexNet	98.53%	98.41%	98.50%	98.45%	00:09:26
InceptionV3	98.61%	98.61%	98.55%	98.58%	01:05:51

## Ethics Statement

This study did not conduct experiments involving humans and animals.

## CRediT Author Statement

**Mei-Ling Huang:** Conceptualization, Methodology, Writing- Original draft preparation, Investigation, Supervision,Writing- Reviewing and Editing, Funding acquisition; **Yi-Xuan Xu:** Conceptualization, Methodology, Writing- Original draft preparation, Software, Data curation; **Yu-Chieh Liao:** Software, Formal Analysis, Data curation.

## Declaration of Competing Interest

The authors declare that they have no known competing financial interests or personal relationships that could have appeared to influence the work reported in this paper.

## References

[1] Wei X., Wang X., Gao Z., Cao P., Han J. (2019). Identification of flower herbs in Chinese pharmacopoeia based on DNA barcoding. Chinese Herb. Med..

[2] Yuan H. (2020). The flower head of Chrysanthemum morifolium Ramat. (Juhua): a paradigm of flowers serving as Chinese dietary herbal medicine. J. Ethnopharmacol..

[3] Grzeszczuk M., Stefaniak A., Meller E., Wysocka G. (2018). Mineral composition of some edible flowers. J. Elem..

[4] Rop O., Mlcek J., Jurikova T., Neugebauerova J., Vabkova J. (2012). Edible flowers - a new promising source of mineral elements in human nutrition. Molecules.

[5] Kumari P., Ujala, Bhargava B. (2021). Phytochemicals from edible flowers: opening a new arena for healthy lifestyle. J. Funct. Foods.

[6] Jiang F., Lu Y., Chen Y., Cai D., Li G. (2020). Image recognition of four rice leaf diseases based on deep learning and support vector machine. Comput. Electron. Agric..

[7] Nilsback M-E., Zisserman A. (2008). 2008 Sixth Indian Conference on Computer Vision, Graphics & Image Processing.

[8] [Dataset] M-L. Huang, Y-X. Xu, (2021), “Chinese medicinal blossom-dataset”, Mendeley Data, V1, doi: 10.17632/r3z6vp396m.1

[9] Shorten C., Khoshgoftaar T.M. (2019). A survey on image data augmentation for deep learning. J. Big Data.

[10] Krizhevsky A., Sutskever I., Hinton G.E. (2012). the Proceedings of the 25th International Conference on Neural Information Processing Systems - Volume 1.

